# Molecular complex detection in protein interaction networks through reinforcement learning

**DOI:** 10.1186/s12859-023-05425-7

**Published:** 2023-08-02

**Authors:** Meghana V. Palukuri, Ridhi S. Patil, Edward M. Marcotte

**Affiliations:** 1 Department of Molecular Biosciences, Center for Systems and Synthetic Biology, University of Texas, Austin, TX 78712 USA; 2 Oden Institute for Computational Engineering and Sciences, University of Texas, Austin, TX 78712 USA; 3 Department of Biomedical Engineering, University of Texas, Austin, TX 78712 USA

**Keywords:** Community detection, Reinforcement learning, Protein complex, Protein interactions

## Abstract

**Background:**

Proteins often assemble into higher-order complexes to perform their biological functions. Such protein–protein interactions (PPI) are often experimentally measured for pairs of proteins and summarized in a weighted PPI network, to which community detection algorithms can be applied to define the various higher-order protein complexes. Current methods include unsupervised and supervised approaches, often assuming that protein complexes manifest only as dense subgraphs. Utilizing supervised approaches, the focus is not on *how* to find them in a network, but only on learning *which* subgraphs correspond to complexes, currently solved using heuristics. However, learning to walk trajectories on a network to identify protein complexes leads naturally to a reinforcement learning (RL) approach, a strategy not extensively explored for community detection. Here, we develop and evaluate a reinforcement learning pipeline for community detection on weighted protein–protein interaction networks to detect new protein complexes. The algorithm is trained to calculate the value of different subgraphs encountered while walking on the network to reconstruct known complexes. A distributed prediction algorithm then scales the RL pipeline to search for novel protein complexes on large PPI networks.

**Results:**

The reinforcement learning pipeline is applied to a human PPI network consisting of 8k proteins and 60k PPI, which results in 1,157 protein complexes. The method demonstrated competitive accuracy with improved speed compared to previous algorithms. We highlight protein complexes such as C4orf19, C18orf21, and KIAA1522 which are currently minimally characterized. Additionally, the results suggest TMC04 be a putative additional subunit of the KICSTOR complex and confirm the involvement of C15orf41 in a higher-order complex with HIRA, CDAN1, ASF1A, and by 3D structural modeling.

**Conclusions:**

Reinforcement learning offers several distinct advantages for community detection, including scalability and knowledge of the walk trajectories defining those communities. Applied to currently available human protein interaction networks, this method had comparable accuracy with other algorithms and notable savings in computational time, and in turn, led to clear predictions of protein function and interactions for several uncharacterized human proteins.

**Supplementary Information:**

The online version contains supplementary material available at 10.1186/s12859-023-05425-7.

## Background

Protein–protein interactions (PPIs) are essential to nearly all cellular functions and biological processes. From antibodies binding antigens to block infections, to protein filaments comprising cellular cytoskeletons, protein interactions are an important organizational principle across biological scales and organisms. Such multi-protein complexes may additionally bind other molecules, such as DNA, RNA, or metabolites, and play critical roles in cellular processes ranging from DNA replication to transcription, multicellular interactions, and tissue organization.

As a consequence, a growing variety of experimental techniques have been developed to determine PPIs at a large scale, notably including affinity purification/mass spectroscopy (AP/MS), co-fractionation/mass spectrometry (CF/MS), cross-linking/mass spectrometry (XL/MS), proximity labeling, and yeast two-hybrid assays (Y2H), which are collectively reviewed in [1–5]. The resulting PPIs define (often weighted) networks of interactions, in which each node represents a protein, an edge represents the interaction confidence, and certain proximal groups of nodes and edges correspond to multiprotein complexes. Importantly, the experimental methods are not completely accurate and suffer both false positive and negative observations. Hence, integrating PPIs across multiple experiments, *e.g.* the networks hu.MAP 1.0 [6] and hu.MAP 2.0 [7] that integrate over 9000 and 15000 mass spectrometry experiments respectively from AP/MS [8–11] and CF/MS data [12–15], can help to mitigate the effects of experimental errors. Combining such approaches with algorithms to cluster proteins and identify complexes from the PPI network should result in a more accurate determination of protein complexes. Community detection algorithms can be applied to a PPI network to identify its communities, *i.e.*, protein complexes [16].

Community detection methods can be unsupervised, *i.e.*, not use any information from known communities in a network and instead rely only on the network topology to cluster it into its communities. Currently, existing unsupervised community detection algorithms tend to rely on many assumptions regarding the topological structures of communities. MCODE (Molecular COmplex DEtection) is an unsupervised method of detecting protein complexes running on the assumption that dense regions of a network represent complexes [17]. Another unsupervised algorithm, CMC (cluster-based on maximal cliques), assumes that communities are mainly in the shape of cliques (again, highly dense subgraphs) [18]. This pattern of similar assumptions carries on to other unsupervised methods such as COACH (core-attachment-based method) [19], ClusterONE (clustering with overlapping neighborhood expansion) [20], and GCE (greedy clique expansion) [21], among others.

The FCAN-MOPSO algorithm is an enhanced clustering method that optimizes communities based on fuzzy clustering logic and multi-objective particle swarm optimization (MOPSO) techniques [22]. It clusters complexes by assigning membership degrees to nodes, allowing for soft assignments, and captures the overlapping nature of clusters. However, there are disadvantages to this algorithm, including computational complexity, sensitivity to parameter settings, and limited applicability to datasets where clusters are expected to be distinct and non-overlapping. Another current community detection algorithm applies the alternating direction method of multipliers (ADMM) to find complexes in a parallel manner [23]. The algorithm decomposes the protein complex detection task into subtasks and applies the ADMM framework to find complexes based on topological and biological assumptions. Though this is computationally efficient, the use of assumptions limits the finding of new protein complexes. HiSCF leverages higher-order structures in biological networks by considering higher-order relationships among nodes and identifies clusters that capture complex patterns and functional modules [24]. The algorithm utilizes Markov clustering (MCL) and iteratively updates and refines clusters based on the expanding inflate operation until groups are well-defined. Other existing algorithms are generally limited to handling only specific connectivity patterns, however, HiSCF was designed to target a wider range of possible patterns [24]. However, HiSCF may face challenges when applied to large interaction networks due to memory requirements and computational complexity. Recent unsupervised algorithms include PC2P, which uses a greedy approximation algorithm based on biclique subgraph properties [25], and MP-AHSA which uses a fitness function for biological similarities within complexes and optimizes a core-attachment-based algorithm for complex identification [26]. Another method, DPCMNE recursively compresses PPI networks, learns multi-level protein embeddings, and applies a core-attachment approach based on the embeddings’ similarities [27].

On the other hand, supervised community detection methods do consider different topological features of communities apart from density, and learn a community fitness function (*i.e.*, the probability of being a community) from known complexes using different learning algorithms. One such approach uses a support vector machine (SCI-SVM) and a Bayesian network (SCI-BN) [28]. For both models, subgraphs are represented using 33 features, and a local subgraph growth process is employed starting from a seed node, with the subgraph growth regulated by limited growth rounds, score improvement over iterations, and extent of overlap with other candidate communities. ClusterSS, a cluster with supervised and structural information, is a supervised algorithm using a neural network, 17 subgraph features, and a structural scoring function [29]. All three methods, SCI-SVM, SCI-BN, and ClusterSS use a greedy heuristic algorithm for selecting the neighbor to add to the subgraph in the growth process, with ClusterSS considering only the top neighbors by degree for speed improvements. However, since the methods use serial candidate community sampling, this negatively impacts their scalability to large networks like hu.MAP 1.0 [6] with ~ 8k proteins and ~ 60k interactions, and hu.MAP 2.0 [7] with ~ 10k proteins and over 40k interactions. To combat this, Super.Complex (supervised complex detection algorithm) was developed for high scalability and accuracy [30]. By using AutoML (Automated Machine Learning), it explores different supervised algorithms to utilize the optimal one learned from known communities. Then, it samples candidate subgraphs using the learned fitness function by growing them with an epsilon-greedy heuristic, along with one of four additional heuristics (pseudo metropolis, clique—pseudo metropolis, iterative simulated annealing, and greedy). However, the AutoML pipeline can take a long computation time and the method can still be improved in terms of accuracy.

While current supervised learning methods learn community fitness functions, they do not learn trajectories on the network that can lead to a protein complex, potentially missing complexes that cannot be traversed using the heuristics employed, for instance in a greedy setting where the community fitness function is maximized at each step. We can apply reinforcement learning to learn paths traversed on a graph to recall known complexes with high accuracy. MARL (Multi-Agent Reinforcement Learning) uses Q-learning to form clusters in networks based on a multi-agent environment [31]. In this algorithm, each node is viewed as an agent and each agent chooses actions to grow into a cluster. A team reward is given to train the action-value function, based on the modularity of the partition, which again assumes that all communities are dense subgraphs. The team reward can also lead to unstable learning of each agent’s behaviors, apart from taking a long time to compute. Nevertheless, MARL has suggested that there is a lot of potential for the use of reinforcement learning in community detection algorithms.

Rather than using a multi-agent approach with a team reward based on modularity, an unsupervised measure, we use a single-agent approach (where a subgraph is viewed as an agent) with a supervised reward from training communities based on whether the agent selects the right neighbor to grow the subgraph. The rewards are used to train a value iteration algorithm to learn an optimal value function that is used to predict new communities. During the prediction phase, we implement a parallel method with a single agent on each core (process) to increase the speed of the algorithm by predicting communities in a distributed fashion. Applying the reinforcement-learning (RL) algorithm on a human PPI network after learning from experimentally characterized complexes, we can identify candidate protein complexes, and these candidates can then be experimentally characterized. We can create reliable models of protein complexes that allow us to extract more information about their stability, affinity, and specificity.

In the current work, we formulate community detection as a reinforcement learning task and implement a value iteration algorithm, learning from known communities. The RL algorithm accurately and efficiently predicts candidate complexes by learning and using a value function from known communities, which maps the density of a subgraph to the probability (score) that traversing the subgraph will yield a protein complex. The algorithm trains on known complexes that have nodes and edges from the network, to accurately optimize scores for the various densities that could occur on the network. Then, the RL pipeline uses these scores to traverse the network by starting with different seed nodes to create candidate complexes in parallel.

The RL pipeline for community detection presents various advantages when compared to its unsupervised and supervised counterparts. Unlike unsupervised methods that rely on assumptions regarding communities such as ‘communities are dense subgraphs of a network’, the RL algorithm takes a more flexible approach by learning trajectories on the network to find known communities having different topologies. To sample candidate communities from the network (an NP-hard problem), while supervised methods have previously used pre-defined heuristics, the RL algorithm learns the correct heuristic to use. Compared to supervised machine learning methods such as neural networks and AutoML methods which require sufficient hyper-parameter tuning and have high training time, the RL pipeline uses the simple value iteration algorithm with few parameters, achieving comparable results in a fraction of the time. Combined with its parallel implementation utilizing multiple cores, the RL pipeline is a fast community detection algorithm enabling efficiency and scalability to large networks. In this paper, we demonstrate the algorithm’s utility by applying it to a high-quality human PPI network (consisting of 8k proteins and 60k pairwise protein interactions) and learn 1,157 candidate protein complexes. These candidates include complexes containing uncharacterized proteins, for which we can suggest possible functions based on ‘honor by association’ with their co-complex members.

## Methods

### Reinforcement learning

Reinforcement learning (RL) uses machine learning to enable an agent to make a sequence of decisions based on rewarding desired behaviors and punishing undesired ones. These rewards reinforce the right decisions so that the agent repeats them. Over time, the algorithm finds the best possible decision or action to take in each situation. Thus, RL is an intuitive method for community detection, as decision-making for the long-term goal of finding a protein complex is needed during the process of traversing a network to grow a complex from a node.

There are three main variables in RL: a state, value function, and reward. A state is a “position” the agent is in, in an environment. A value function is a score given to a state that estimates how beneficial it is for the agent to be in that state to achieve the goal. Lastly, a reward is an incentive that tells the agent if the decision made was correct or not. For example, consider a computer playing against a human in a game of tic tac toe. The AI player is an *agent* created to perform certain *actions* on the tic tac toe grid (the *environment*) based on what *state* the environment is in, in real-time. After each action taken, the agent receives a *reward* based on the four possible outcomes of what the *state* could result in: it wins, the opposing player wins, a draw, or continues the game. If the agent wins, it will receive a positive reward (*i.e.*, 1), and if the opposing player wins or if there is a draw—it will receive a negative reward (*i.e.*, -1), and if the game continues there will be no reward (*i.e.*, 0 or None). The AI player continues to make moves based on what *state* it currently is in to maximize its cumulative reward or *return*. This cycle, also known as an *episode*, terminates when the game ends. While trying to maximize its rewards, the agent will learn the optimal policy, *i.e.*, the best action to be taken in a particular state. The optimal policy is learned by starting with an initial policy and adapting it based on its experience encountering various states.

#### Value iteration

The optimal policy at a state is performing an action that takes the agent to the next best state that will maximize the probability of achieving the goal. In other words, the optimal policy, out of the states available, moves the agent to the state having the highest value function. The true or optimal value function, *i.e.*, a map of the states to their values can be learned using the value iteration algorithm, a classic reinforcement learning method for problems where a model of the environment dynamics is known, usually with a small number of discrete states. The algorithm is a dynamic programming method that solves the Bellman Optimality Equation (Eq. 1) iteratively, converging to the optimal value function V*.

1 $$V^{*} \left( {s_{t} } \right) = \max_{{a_{t} }} \left( {\Sigma_{{s_{t + 1} }} p(s_{t + 1} | s_{t} , a_{t} )} \left( {R\left( {a_{t} , s_{t} } \right) + \gamma V^{*} \left( {s_{t + 1} } \right)} \right)\right)$$

Here, *R* is the defined reward associated with performing an action *a*_*t*_ when the state is *s*_*t*_, *γ* is a discount factor and *p* is the transition probability from *s*_*t*_ to *s*_*t*+*1*_ when it performs action *a*_*t*_.

The value iteration update rule, starting with a value of 0 for all states is given by Eq. (2):

2 $$V_{k} \left( {s_{t} } \right) = \max_{{a_{t} }} \left( {\Sigma_{{s_{t + 1} }} p(s_{t + 1} | s_{t} , a_{t} )\left( {R\left( {a_{t} , s_{t} } \right) + \gamma V_{k - 1} \left( {s_{t + 1} } \right)} \right) } \right)$$

Here*, V*_*k*_* (s*_*t*_*)* is the value function of the current state (at time* t* in the episode) in the current iteration *k* (of the value iteration algorithm) and* V*_*k-1*_* (s*_*t* +*1*_*)* is the value function (from the previous iteration *k-1*) of the next possible state.

### Formulating community detection as a reinforcement learning problem

To build an RL pipeline for the problem of community detection, the algorithm is first trained on known training communities or complexes. Once the training is deemed to be successful, the learned value function from the training is then used to find complexes on a network.

To learn the value function, each episode consists of starting with a seed node from a training complex and iteratively adding neighbors to grow the subgraph into the complex. This process is then repeated with a new seed node from the training complex. Once all the nodes of the complex have been used as seeds, training moves to the next complex. In this scenario, the agent and environment are defined as the current subgraph in the growth process and the full graph including all its neighbors, respectively. We represent the state of the agent, *i.e.*, the current subgraph by its topological feature, density. The state (density *d*) of the current subgraph is the ratio of the actual number of edges in a subgraph to the total possible number of edges and can be calculated with Eq. (3).

3 $$d = \frac{2m}{{n\left( {n - 1} \right)}}$$

Here *m* is the sum of the edge weights of the edges in the subgraph and *n* is the number of nodes in the subgraph.

For a wide representation of the feature space, the states are discretized into 20 intervals ranging from 0 to 1. The actions performed by the agent comprise adding a neighbor to the current subgraph or terminating the growth process. Choosing a neighboring node in the known complex will provide the agent with a positive reward of + 0.2, and a negative reward of -0.2 is given if the node chosen is not present in the known complex. The rewards aid the agent in avoiding previous mistakes for it to find an optimal path to create a complex. These rewards allow the state to develop a value function representing the probability of the state resulting in a final community. If none of the remaining neighbors are in the complex, the agent is encouraged to learn to terminate the growth process by receiving a reward of 0, as opposed to choosing a neighbor giving a reward of − 0.2.

Once the training completes and an optimal value function is learned, the agent learns candidate complexes on the entire network by starting with seed nodes in parallel and adding neighbors giving the highest value function at each iteration, until the action of terminating a growth process gives a higher value than adding any of the neighbors.

#### Proof of applicability of RL to community detection

The environment is deterministic and the next state the subgraph moves into, (*s*_*t*+*1*_), is only dependent on the previous state (*s*_*t*_), the current subgraph. It does not depend on any other states previously encountered by the agent, satisfying the Markov property (Eq. 4) with a memoryless process.

4 $$p(s_{t + 1} |s_{t} ) = p(s_{t + 1} |s_{t} ,s_{t - 1} ,... s_{0} )$$

Here, on the left-hand side, *p* is the conditional probability of achieving a state given only the previous state, while on the right-hand side, the probability is conditional on all the previous states encountered. Therefore, with this formulation, community detection can be treated as a Markov Decision Process (MDP) and solved using reinforcement learning methods.

#### Value iteration for community detection

The value iteration update rule with our formulation of the community detection problem is given by Eq. (5):

5 $$V_{k} \left( {s_{t} } \right) = max_{{a_{t} }} \left( {R\left( {a_{t} , s_{t} } \right) + \gamma V_{k - 1} \left( {s_{t + 1} } \right)} \right)$$

*V*_*k*_* (s*_*t*_*)* is the value function of the current state (at time *t* in the episode) in the current iteration *k* (of the value iteration algorithm), *R* is the defined reward, γ is the discount factor (0.5), and* V*_*k-1*_* (s*_*t* +*1*_*)* is the value function (from the previous iteration *k-1*) of the next possible state. We obtain this simple update rule, derived from the Bellman optimality equation (Eq. 6) using a transition probability *p*(*s*_*t*+*1*_|*s*_*t*_*, a*_*t*_) of 1 in Eq. (1) due to the deterministic nature of this problem, *i.e.*, the state transitions from *s*_*t*_ to only *s*_*t*+*1*_ when action *a*_*t*_ is taken.

6 $$V^{*} \left( {s_{t} } \right) = max_{{a_{t} }} \left( {R\left( {a_{t} , s_{t} } \right) + \gamma V^{*} \left( {s_{t + 1} } \right)} \right)$$

Here, V^*^ is the optimal value function, which the algorithm’s value function converges to after a few iterations using the value iteration update rule (Eq. 5), starting with a value of 0 for all states.

### Reinforcement learning community detection algorithm

#### Overview

There are three main steps for community detection on a network using reinforcement learning:

1. *Training* the algorithm to walk across training complexes by learning a value function corresponding to each state (subgraph) encountered in the process.
2. *Finding* candidate complexes by using the learned value function to walk on the protein–protein interaction network.
3. *Benchmarking* learned complexes against known complexes.

#### Training the algorithm

**Fig. 1:**
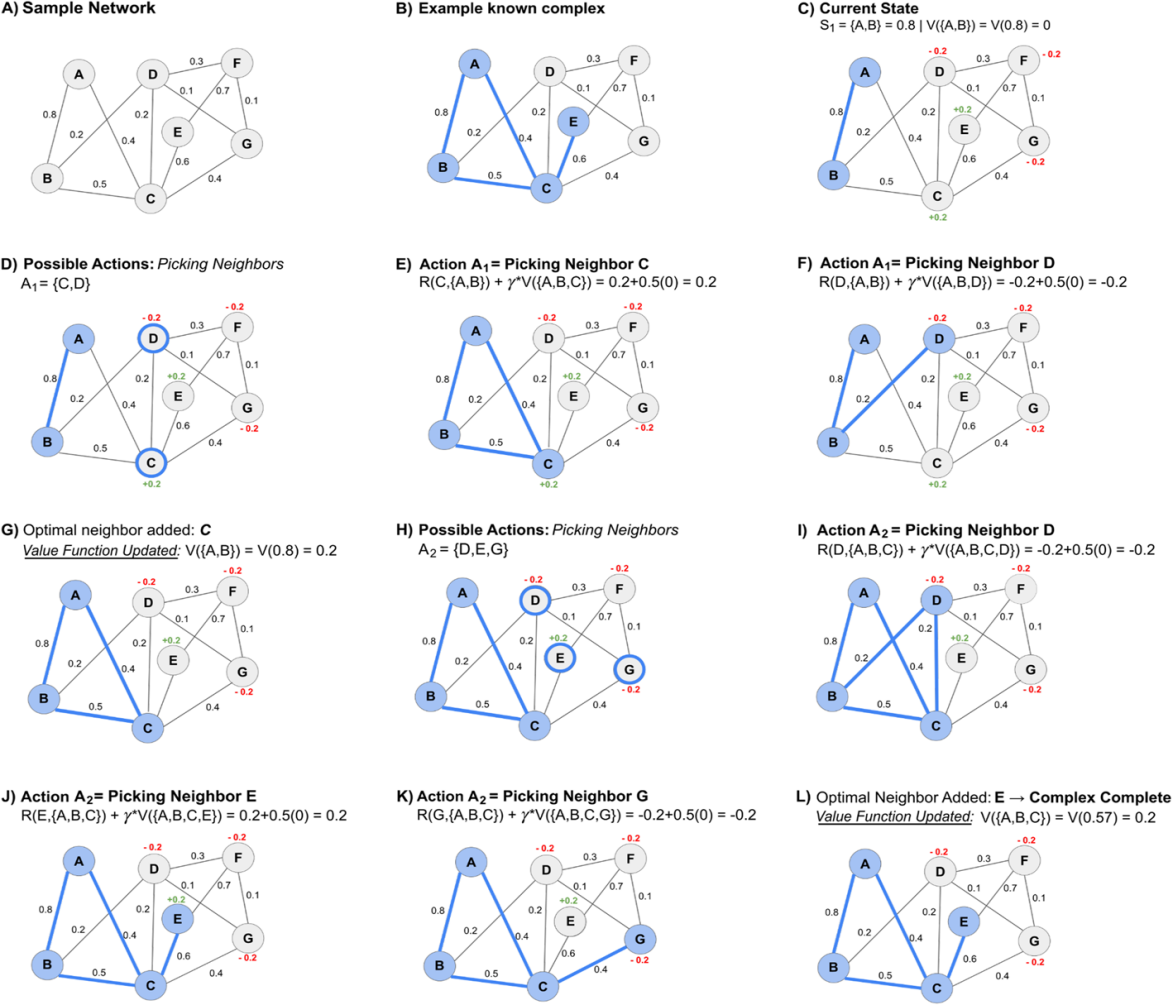
Example trajectory of training the RL pipeline on a network by learning a value function. **A** This network comprises 7 nodes and 11 weighted edges **B** A known complex consists of the nodes A, B, C, and E. **C** First, a seed edge (A, B) is identified, where the state (density) is S_1_ = 0.8 and the value function is V(0.8) = 0 (all densities are initialized to 0). Once a node is added, a reward of + 0.2 is given if the node is in the training complex and − 0.2 if absent. **D** We evaluate all possible neighbors i.e., C and D, to add to the current subgraph {A,B}. Using the value iteration update rule (with γ = 0.5), we compute a corresponding value for the current state by adding each neighbor. **E** Adding node C updates V(0.8) = 0.2. **F** Adding node D updates V(0.8) = − 0.2. **G** The neighbor providing the highest value function (C) is added to the candidate complex and the original state’s value function S_1_{A,B} = 0.8 is now + 0.2. **H** Again, we evaluate all possible neighbors of the updated complex S_2_{A,B,C} = 0.57, i.e., D, E, and G. **(I)** Node D updates V(0.57) = − 0.2.** J** Node E updates V(0.57) =  + 0.2.** K** Node G updates V(0.57) = − 0.2.** L** Node E is added to the complex and V(0.57) is updated to + 0.2. This process is repeated until growth termination by adding an imaginary node with reward 0. As the remaining neighbors D, F, and G have a reward of − 0.2, the imaginary node is chosen as it results in the highest value function (0.1). The candidate complex is then finalized. A new seed edge is chosen from the network and this process repeats

**Fig. 2:**
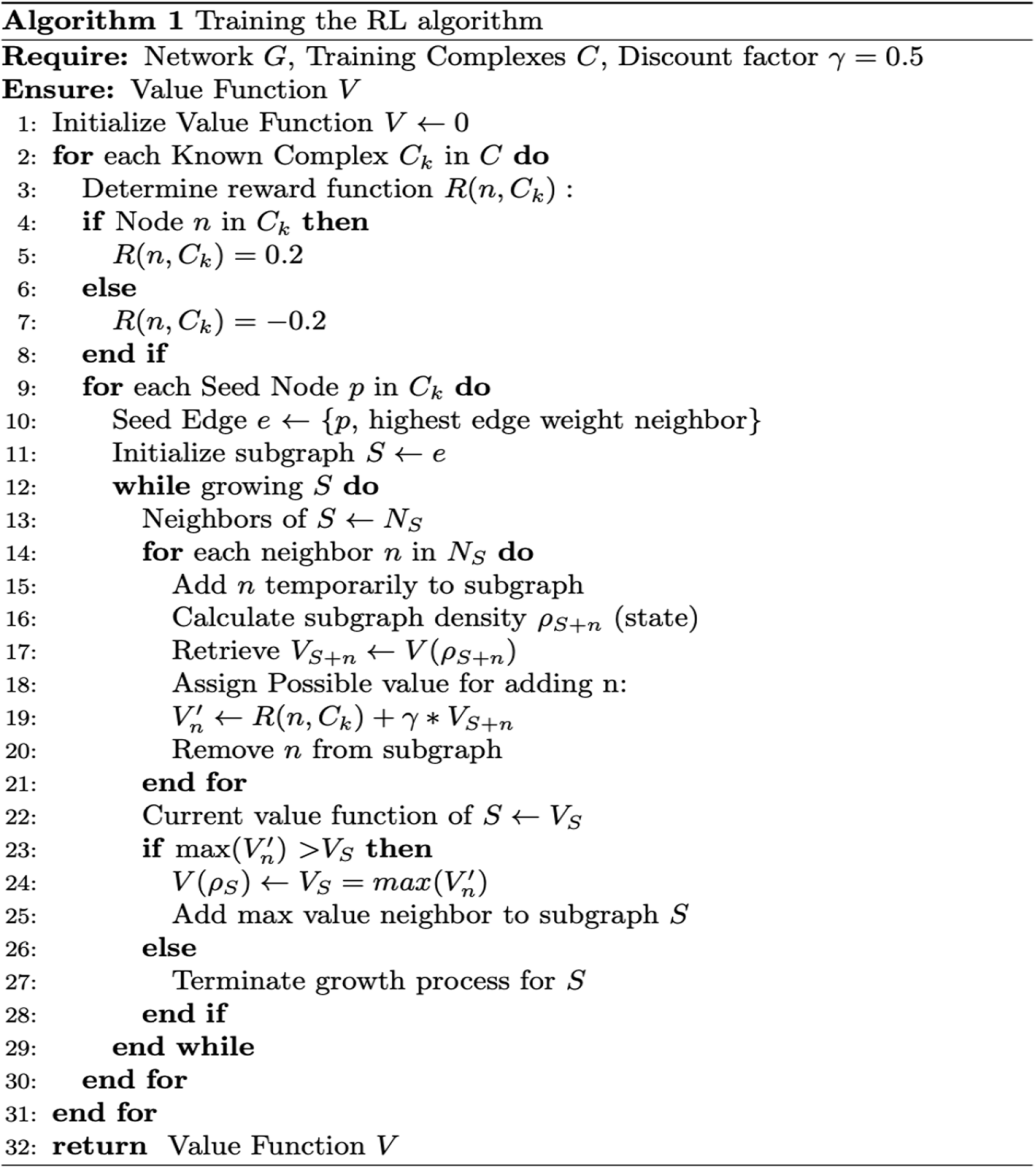
Algorithm 1 outlines the training process for the RL algorithm

For training the RL pipeline, we use the known training complexes and represent each complex as a target subgraph of the protein-interaction network. The agent, *i.e.*, the current subgraph expands by adding, at each step, the neighbor that yields the highest value for the current subgraph (the value is calculated using the reward given for adding this node and the value of the next state, as shown in Eq. 5). The algorithm updates the value of the density of the current subgraph to this new value (Eq. 5). Each time a state (density) is encountered in the process of training on multiple training complexes, the value of that state is updated using the update rule (Eq. 5), moving towards convergence of the value function and eventually learning a value function mapping densities to their probability of leading to a protein complex. Figure 1 shows an example of learning the value function while traversing a single known complex, and Algorithm 1 (Fig. 2) summarizes the training procedure. The initial subgraph or seed is an edge between an initial random node of the complex and the node’s neighbor with whom the node shares an edge with the highest edge weight. To calculate the potential value of the current subgraph, *i.e.*, the term within the max () in Eq. 5 for each neighbor of the current subgraph, each neighbor is temporarily added to the subgraph. The density of that temporary subgraph is calculated, followed by querying the value function for that state along with the reward based on whether the neighbor is present in the final protein complex. After calculating the potential value function, the neighbor is removed from the subgraph for this process to be repeated for the rest of the neighbors. Once all the neighbors have been evaluated, the value function of the current state is updated and the neighbor yielding the state that provided the maximum value function is added to the subgraph. This new subgraph will be the new "seed" as this process repeats itself. The subgraph, or complex, will be "complete" and the algorithm stops adding nodes if all the neighboring nodes return lower value functions than the action of terminating the growth process, represented by adding an imaginary node with reward 0, leading to the same state as before, indicating that no new neighbors should be in the complex. Note how a reward of 0 encourages the algorithm to terminate the growth process when all other options are adding wrong nodes, *i.e.*, choosing actions that have a reward of -0.2. Conversely, if a correct node is available, its reward of 0.2 encourages choosing that node over terminating growth. Once a subgraph is deemed as complete, the process is repeated starting with a different node of the same complex, so that other trajectories to build the same complex are explored and the value function is updated accordingly. After starting with each of the nodes of a complex as seeds, training moves to the next complex, starting with a random new seed from this complex, and the process repeats.

#### Finding candidate complexes

**Fig. 3:**
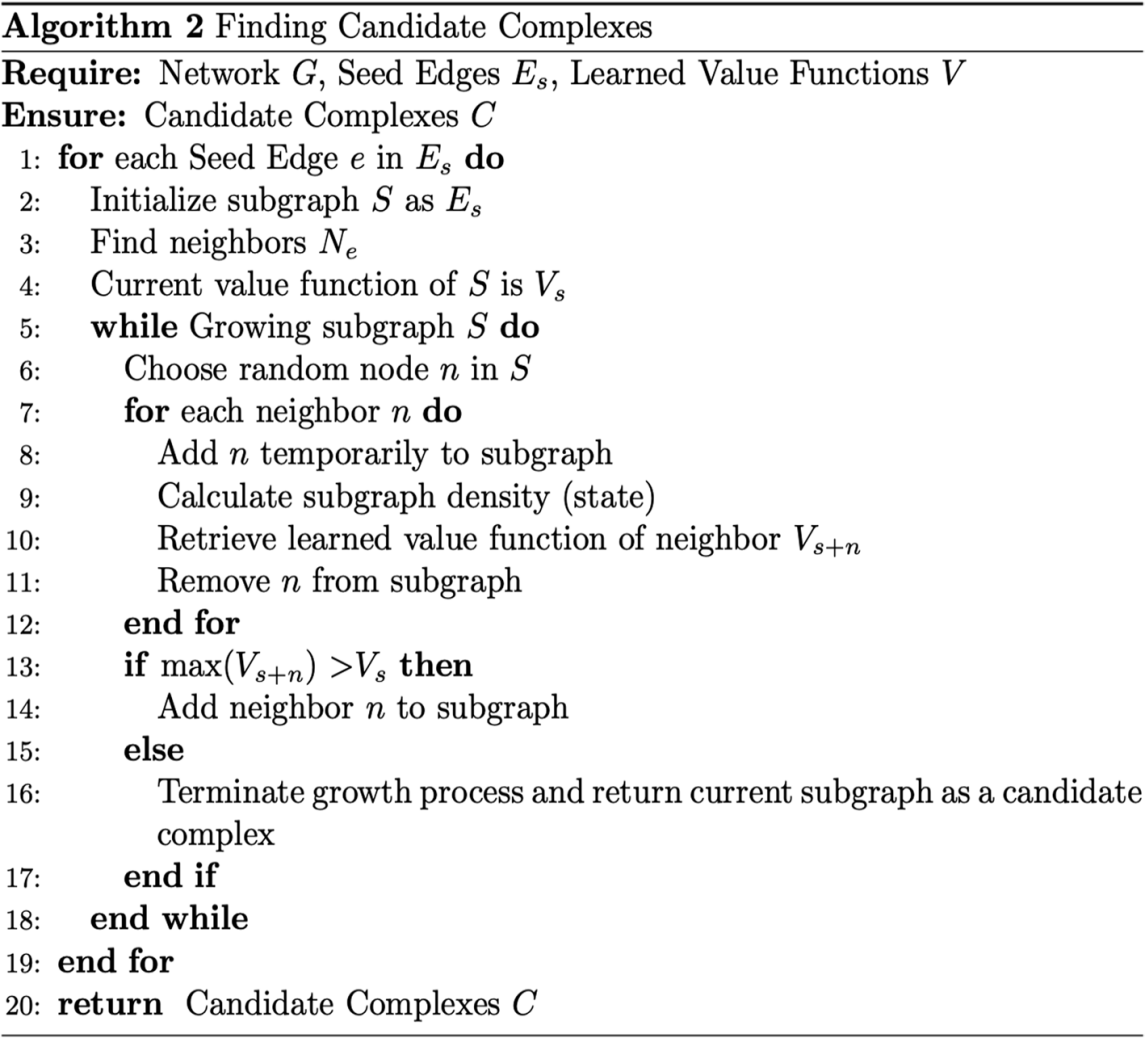
Algorithm 2 outlines the steps in the RL algorithm to identify candidate complexes

**Fig. 4:**
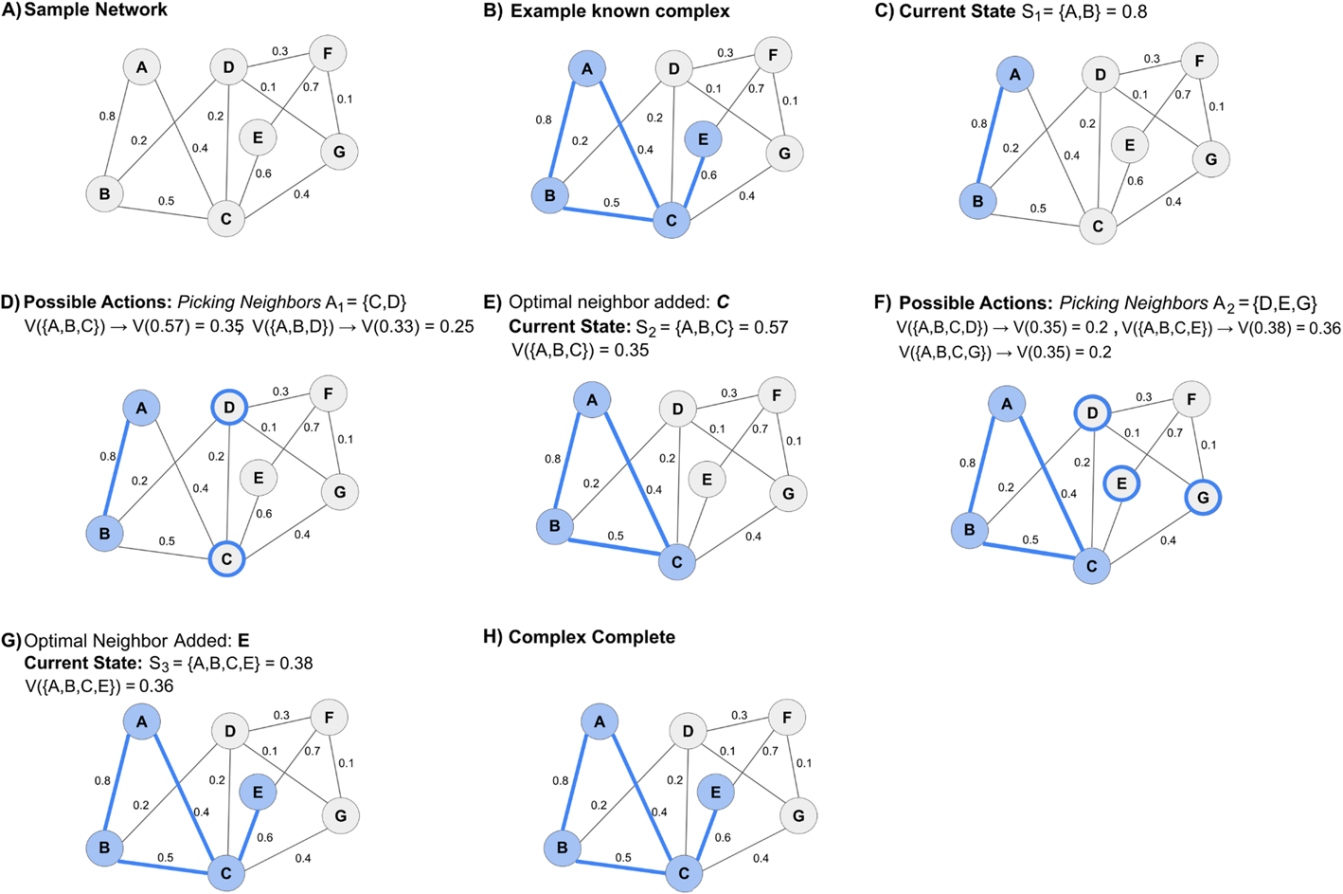
Example trajectory for finding a complex with the RL pipeline using a learned value function. **A** This network comprises 7 nodes and 11 edges, each with corresponding with an edge weight. **B** In this network, a known complex consists of the nodes A, B, C, and E. The goal is to predict this known complex from the network using the learned value function. **C** A seed edge is identified to begin the walk (edge AB). At this seed edge, the complex is at state (density) S_1_ = 0.8. **D** Then, we evaluate all possible neighbors of nodes A and B, i.e., C and D. Adding node C gives a temporary complex {A, B, C} with S_2_ = 0.57, and a learned value V({A, B, C}) = 0.35, while adding node D gives a temporary complex {A, B, D} with S_2_ = 0.33, V({A, B, D}) = 0.25. **E** The neighbor with the highest value function is node C and hence, node C is added resulting in S_2_ = 0.57, V({A, B, C}) = 0.35. **F** The next neighbors are evaluated, i.e., D, E, and G. Adding node D leads to S_3_ = 0.35, V({A, B, C, D}) = 0.2, node E results in S_3_ = 0.38, V({A, B, C, E}) = 0.36, and node G leads to S_3_ = 0.35, V({A, B, C, G}) = 0.2. **G** Since the neighbor yielding the highest value function is node E, this node is added to the complex resulting in S_3_ = 0.38, V ({A, B, C, E}) = V(0.38) = 0.36. **H** Each neighbor (D, F, and G) results in a value function less than the current complex {A, B, C, E}. Thus, no neighbor is added, and the predicted community is complete

Once we observe that the value functions of different states have converged in the training process, we can use the learned value function to walk paths on the network to find complexes. For each node of the network, we choose its corresponding highest edge-weight as a seed edge to grow a candidate complex. For each seed edge, the neighbors are evaluated and the neighbor yielding the subgraph with the maximum value function is added. The growth process stops when adding any neighbor lowers the value function of the current subgraph. For instance, consider a subgraph with a value function of 0.2. If on evaluating each of the subgraph’s neighbors, each of them returns a value function lesser than 0.2, the algorithm terminates, and the subgraph will be considered a candidate complex. This process is repeated for the next seed edge in the network. These steps are detailed in Algorithm 2 (Fig. 3) and an example of finding a complex on the network is shown in Fig. 4.

#### Post-processing and evaluation

Once we have found all the candidate subgraphs corresponding to the specified seed nodes (in our experiments we use all the nodes of the graph as seed nodes), we perform a post-processing step to merge highly overlapping complexes. Adapting the pairwise merging algorithm in Super. Complex [30], if the overlap of two complexes is more than a specified threshold, we retain the complex with the highest value function of the two complexes and the merged variant and remove the others. To obtain the optimal overlap threshold, similar to [30], we test various thresholds for the *Qi overlap* measure (Eq. 7) and choose the threshold which gives the highest F-similarity-based Maximal Matching F-score (FMMF).

7 $${\text{Qi}}\;{\text{overlap}}\;{\text{measure}}:\; \frac{{\left| {C_{p} \cap C_{k} } \right|}}{{|C_{p} |}} > t\;{\text{and}}\; \frac{{\left| {C_{p} \cap C_{k} } \right|}}{{|C_{k} |}} > t$$

Here, *t* is the user-specified overlap threshold, *C*_*p*_ is a predicted complex and *C*_*k*_ is a known complex.

To gauge the accuracy of the RL algorithm, we use different evaluation measures to compare learned complexes with known complexes. The learned complexes are compared with the known complexes after removing nodes missing in the set of known complexes. We employ a variety of evaluation measures such as the FMMF, Community-wise Maximum F-similarity-based F-score (CMMF), and Unbiased Sn-PPV Accuracy (UnSPA) defined in Super.Complex [30], in addition to the Qi et al. F-score [28], F-grand k-clique and F-weighted k-clique [6].

As discussed in [30], one of the more accurate methods for comparison is the FMMF which combines an adapted Maximal Matching Ratio (MMR) with its corresponding precision. The adapted MMR is computed as the fraction of known complexes matching predicted complexes, where the matches are computed as the sum of edge weights of a maximal matching in a bipartite graph between learned and known complexes. The edge weights used in the graph, matching learned and known complexes, are the F-similarity scores (F1) computed as follows.

8 $$p^{\prime} = \frac{{\left| {C_{p} \cap C_{k} } \right|}}{{\left| {C_{p} } \right|}}$$

9 $$r^{\prime} = \frac{{\left| {C_{p} \cap C_{k} } \right|}}{{|C_{k} |}}$$

10 $$\frac{2}{F1} = \frac{1}{p^{\prime}} + \frac{1}{r^{\prime}}$$

Here, *C*_*p*_ is a derived complex and *C*_*k*_ is a known complex.

## Results

To demonstrate the effectiveness of the RL algorithm, we show the algorithm’s performance on synthetic and real datasets and discuss the human protein complexes learned by the algorithm. We first evaluate the algorithm on a synthetic toy dataset to help provide intuition for how the RL process works on a simple system that can be fully visualized. Then, we apply RL to the current full-scale human protein interaction network, focusing here specifically on the discovery of higher-order physical protein assemblies. For this purpose, we analyzed the hu.MAP networks (hu.MAP 1.0 [6] and hu.MAP 2.0 [7]), which were originally developed to maximize the quality and scale of protein interactions derived from mass spectrometry proteomics experiments and which have been independently shown to capture true protein interactions to a significant degree [32, 33]. Finally, we analyzed the set of derived complexes for uncharacterized proteins and complexes.

In this section, we start with the details of experiments carried out, and then examine the value functions learned in these experiments as these are the key part of the algorithm, signifying how to learn trajectories on the network corresponding to communities. Then, we present the evaluation metrics of the experiments including comparisons with state-of-the-art methods. In conclusion, we analyze the derived complexes from the experiment on the human protein interaction network, highlighting the complexes with uncharacterized proteins.

### Experimental details—synthetic and protein interaction datasets

**Fig. 5:**
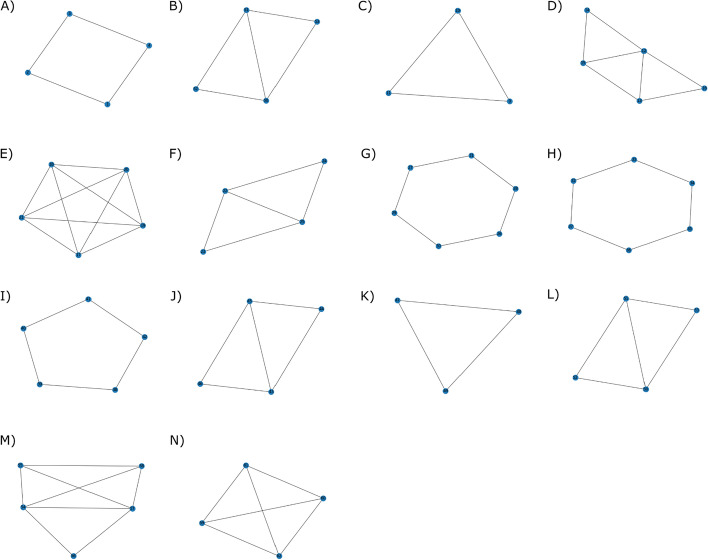
A synthetic disconnected toy network of complexes. Complexes **A**–**G** are used for training and **H**–**N** are used for evaluating the RL algorithm

We first evaluate the RL algorithm on a synthetic toy network (Fig. 5) with 62 nodes and 78 edges, comprising 14 complexes. Of these 14 complexes, 7 are used as training complexes and 7 are used as testing complexes. The testing and training evaluation results can be found in Additional file 1: Table S1. The results showed consistently high scores across various evaluation metrics, indicating strong precision and accuracy in predicting both training and testing toy complexes.

Next, we apply the RL algorithm on a human protein interaction network (hu.MAP 1.0 [6]) comprising 7778 nodes and 56,712 edges to learn candidate complexes using 188 known complexes; these complexes are obtained by pre-processing the CORUM protein complex database [34]. The pre-processing (see methods section of Super.Complex [30]) primarily involves discarding complexes that are internally disconnected or have fewer than 3 nodes. We also merge complexes with a pairwise overlap of more than 0.6 Jaccard coefficient to remove redundancy, since the CORUM set of complexes includes multiple non-independent complexes, e.g., the 19S and 20S proteasomes are subcomplexes of the larger 26S proteasome. We merge these highly interdependent complexes to help reduce ambiguity while training the RL agent. Since we train on only one complex in each episode, reducing non-independent complexes will reduce negative rewards being given when the RL agent adds a neighbor that does not belong to the complex, but belongs to another overlapping complex. We also discuss an alternate reward scheme in the conclusions section for overlapping community detection.

For a perfect comparison, we use the same pre-processing steps as in Super.Complex for both the network and complexes, as well as the same training and testing complexes (all input data was obtained from the Super.Complex input data [30]). Since we only use the density feature in our algorithm, we also run the Super.Complex pipeline with only the density feature. The testing and training evaluation results can be found in Additional file 1: Table S2.

The RL algorithm is also tested on hu.MAP 2.0 [7], a human PPI network consisting of 10,433 nodes and 43,581 edges, obtained by considering only the edges with a weight of at least 0.02. Again, to compare with Super.Complex, we use the same training and testing complexes from hu.MAP 1.0, and the same preprocessing steps. We perform two experiments; in the first experiment, we transfer the value function trained on hu.MAP 1.0 and in the second, we train a new value function on hu.MAP 2.0. The testing and training evaluation results for hu.MAP 2.0 can be found in Additional file 1: Table S3.

### The value function converges in the training phase

**Fig. 6:**
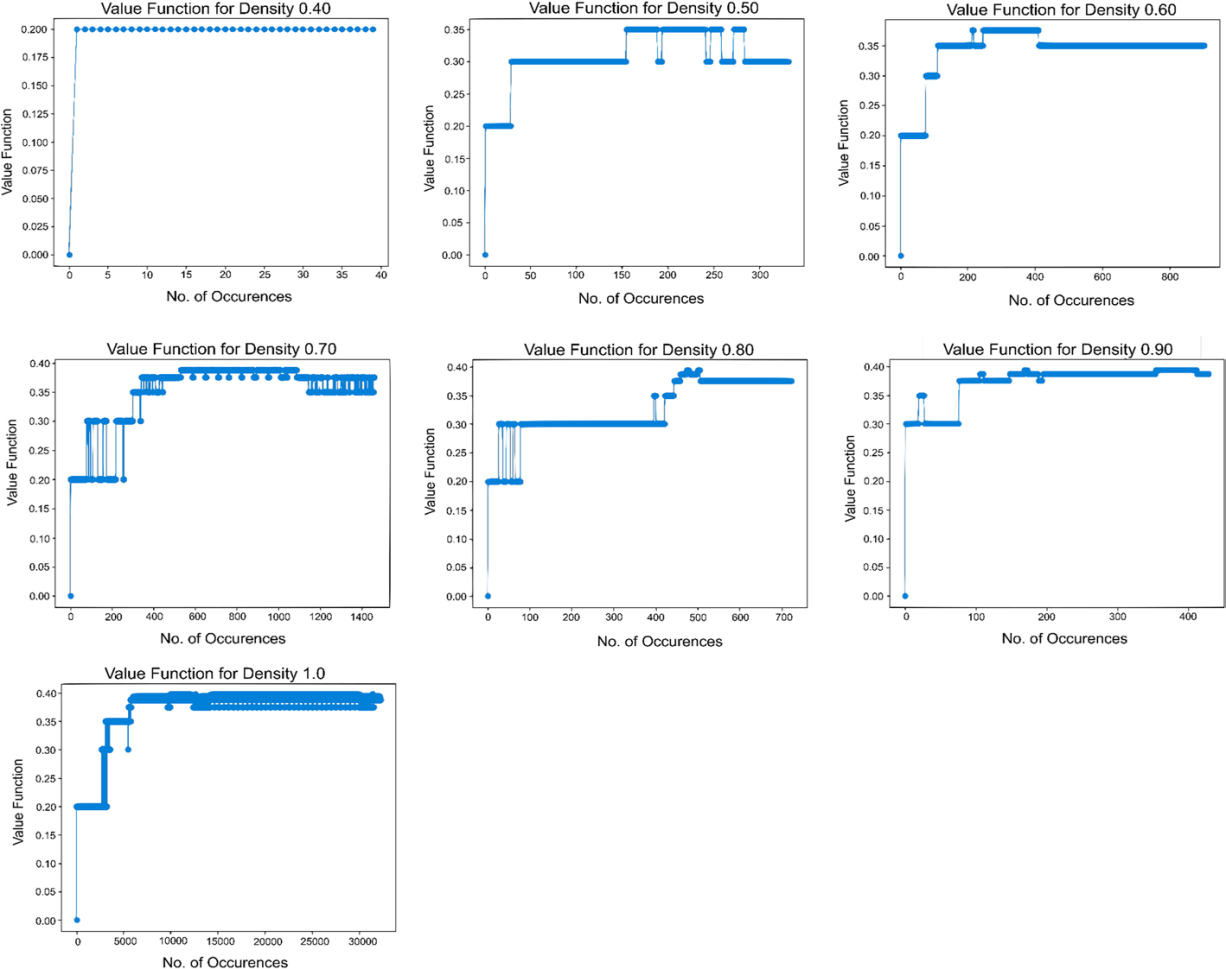
Convergence of the value for each density. Each density (0.4 through 1) encountered in the training for hu.MAP 1.0 is plotted to see how its value updates over iterations. Although the values fluctuate initially, they converge eventually indicating successful training

In this section, we examine the value functions learned in the experiments, as these signal the key heuristic learned to identify trajectories on the network leading to communities. During the training phase, we track the value for each encountered state (density) over time. Once the value of each of the states starts to converge, it can be assumed that the value has reached its optimum. Figure 6 demonstrates the successful convergence of the value for each density in hu.MAP 1.0.

**Fig. 7:**
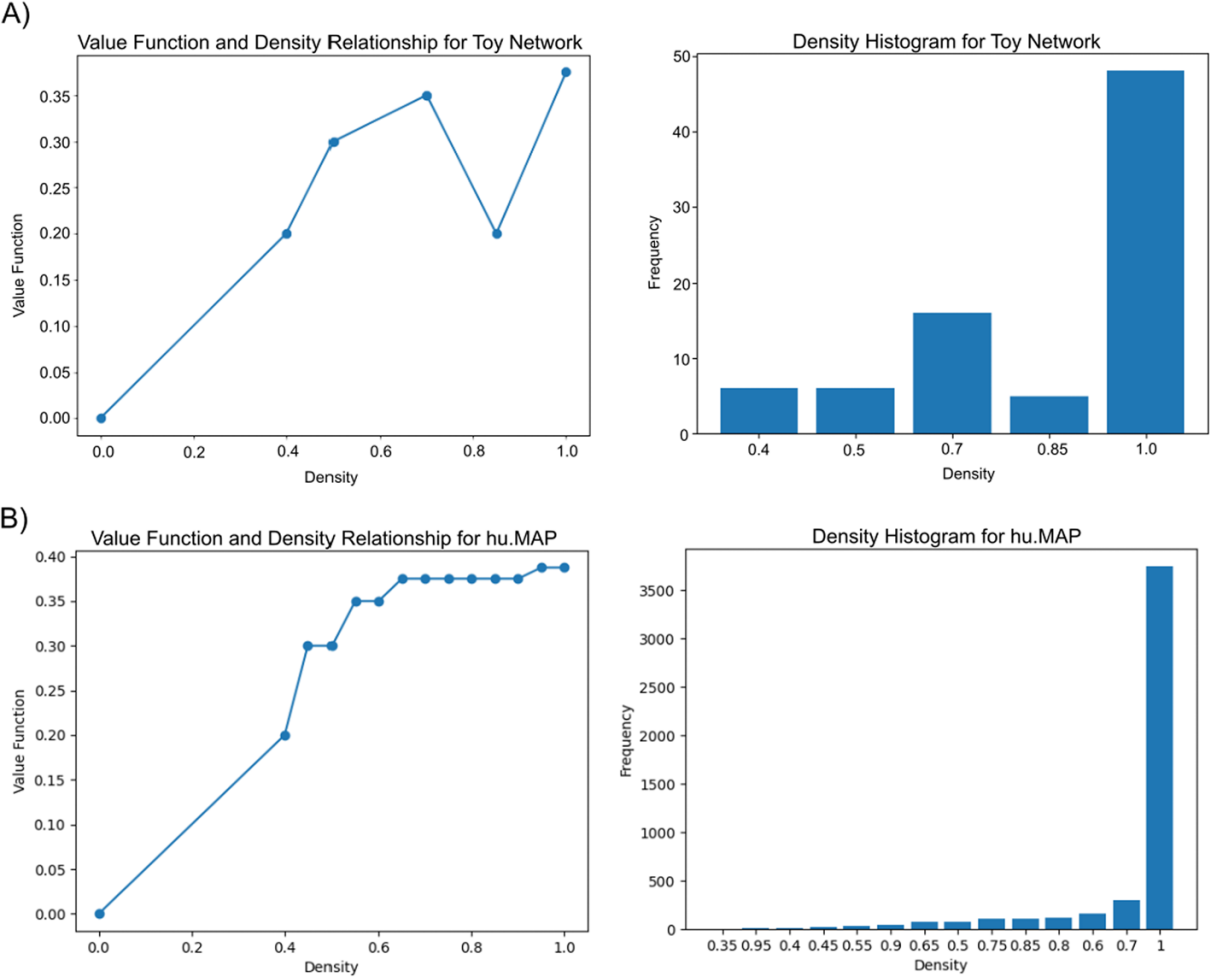
Higher values favor higher densities. **A** The graph on the toy network shows a positive correlation between value function and density and therefore, complexes and trajectories with higher densities are favored. The density histogram for the training phase shows a higher frequency for higher density subgraphs. **B** For hu.MAP 1.0, the graph shows a stronger correlation between density and value function. The density histogram again shows that higher densities are more frequently observed

We also investigate the relationship between a state’s density and its value. Figure 7 shows that on the path to a final complex, subgraphs of higher densities are favored since they have higher values.

The learned value functions for the synthetic dataset and hu.MAP 1.0 enable us to accurately predict complexes on the respective networks, as shown in the next section. Further, employing transfer learning, we use the value function learned on hu.MAP 1.0 to accurately predict complexes on hu.MAP 2.0 (Additional file 1: Table S3). This demonstrates that the value functions learned by the RL algorithm can be transferred for community detection problems on similar networks. We also directly train a value function on hu.MAP 2.0 using the same training complexes and find that predicting complexes on hu.MAP 2.0 with this value function also gives accurate results (Additional file 1: Table S3).

### The RL algorithm learns accurate communities on synthetic and real datasets

**Fig. 8:**
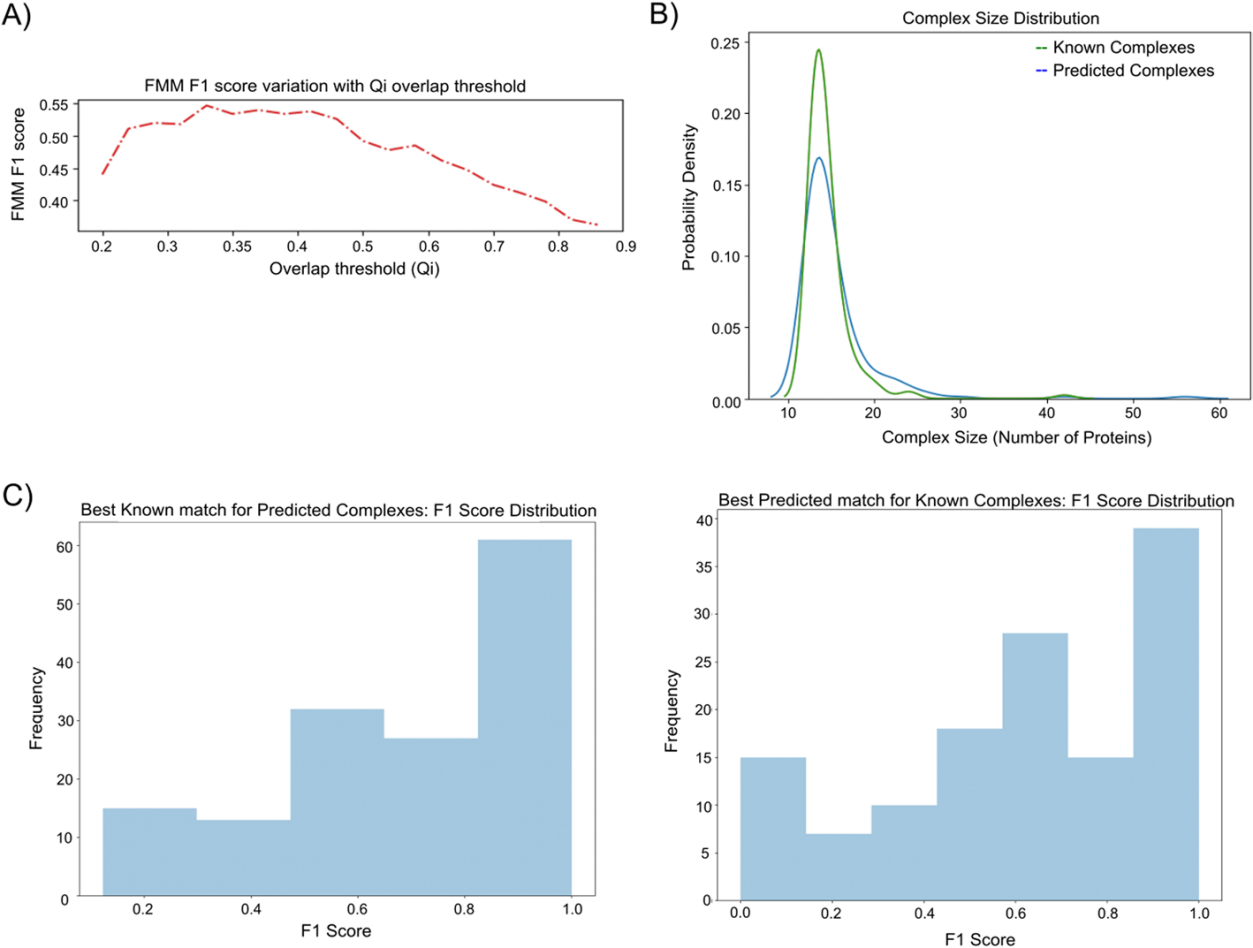
Evaluating the predictions of the RL algorithm on hu.MAP 1.0. **A** The optimal Qi threshold is 0.325. We tested various overlap thresholds, *i.e.*, Qi values (Eq. 7) between 0.2 and 0.9 in 0.25 intervals. **B** Size distributions of known and predicted complexes. This graph shows that the distribution of the sizes (no. of proteins) of the predicted and known complexes is very similar. **C** F1 score distributions of the best-predicted match for known complexes and vice-versa. In both cases, higher F1 scores have higher counts indicating accurate predictions

Having learned accurate values of different states (subgraphs) encountered in the process of reconstructing known communities, we now use the value functions learned to find communities on the whole network. Recall the synthetic dataset containing 14 communities used to evaluate the RL algorithm. The performance of the algorithm across different evaluation measures is excellent as summarized in Table 1. Next, we apply the algorithm to the real dataset, hu.MAP 1.0, by training it on 132 complexes. We test different Qi overlap thresholds (Fig. 8A), in the RL algorithm, to merge highly overlapping complexes. The peak in Fig. 8A occurring at 0.325 Qi overlap measure corresponds to the best FMMF score. For this value of the Qi overlap measure, the RL algorithm learns 1,157 complexes. We also analyze the complex size distribution (Fig. 8B) and examine the best known complex matches for derived complexes and the best derived complex matches for known complexes using F1 score distributions (Fig. 8C).

**Table 1:** RL algorithm has strong performance on a synthetic toy dataset

	FMM Precision	FMM Recall	FMM F-score	CMMF	UnSPA	Qi et al. F1 score	SPA	F-Grand K-Clique	F-weighted K-Clique
RL Algorithm	0.963	0.963	0.963	0.963	0.969	1.00	0.959	1.00	1.00
Super.Complex	0.999	0.999	0.999	1.00	0.999	1.00	0.998	1.00	1.00

The algorithm was trained on 7 toy complexes from a synthetic network of 62 nodes and 78 edges. It predicted 14 complexes which are evaluated against the 14 true complexes

FMM, F-similarity-based Maximal Matching; CMMF, Community-wise Maximum F-similarity-based F-score; UnSPA, Unbiased Sn-PPV Accuracy; SPA, Sn-PPV Accuracy

For a perfect comparison, Super.Complex is evaluated on hu.MAP 1.0 using only the subgraph feature density. The best results from Super.Complex are obtained using a k-nearest neighbors’ classifier (with k = 76) to train a community fitness function, and from a search process for candidate complexes using maximal cliques as starting seeds and a pseudo-metropolis heuristic (with a probability of 0.1) for complex growth (with an exploration probability ϵ of 0.01). The candidate complexes are then merged with an overlap threshold of 0.2 Jaccard coefficient to yield 798 final complexes. In contrast, the RL algorithm predicts a higher number (1157) of complexes possibly explaining the slightly higher (FMM) recall measure (Table 2). The RL method achieves comparable performance to the supervised method, Super.Complex and tends to outperform 4 recent unsupervised community detection methods, ClusterONE + MCL [6], PC2P [25], MP-AHSA [26], and DPCMNE [27] (Table 2), demonstrating the potential of applying reinforcement learning to community detection.

**Table 2:** The RL algorithm yields competitive accuracy compared to other community detection algorithms on hu.MAP 1.0

	FMM Precision	FMM Recall	FMM F-score	CMMF	UnSPA	Qi et al. F1 score	F-Grand K-Clique	F-weighted K-Clique
RL Algorithm	0.612	0.482	0.547	0.654	0.772	0.559	0.789	0.988
Super.Complex	0.835	0.457	0.591	0.720	0.803	0.658	0.991	0.999
ClusterONE + MCL	0.471	0.686	0.579	0.797	0.911	0.794	0.77	0.967
PC2P	0.535	0.589	0.562	0.625	0.571	0.418	0.627	0.891
MP-AHSA	0.421	0.498	0.459	0.424	0.513	0.397	0.516	0.823
DPCMNE	0.457	0.048	0.086	0.408	0.454	0.132	0.609	0.608

The learned complexes on hu.MAP 1.0 are evaluated against all the known cleaned CORUM complexes

FMM, F-similarity-based Maximal Matching; CMMF, Community-wise Maximum F-similarity-based F-score; UnSPA, Unbiased Sn-PPV Accuracy; SPA, Sn-PPV Accuracy

Comparing Tables 1 and 2, we observe better accuracies for the algorithm on the toy dataset than those on hu.MAP 1.0. This could be attributed to the algorithm being better suited to finding non-overlapping communities, such as the toy communities, when compared to finding overlapping communities, such as the CORUM complexes on hu.MAP 1.0. We also evaluated the RL algorithm without merging overlapping communities in the predicted complexes, resulting in an FMM F1-score of 0.102 and a Qi et al. F1-score of 0.255. A comparison with the scores obtained when employing the merging operation (Table 2) reveals a significant improvement in metrics after eliminating overlapping complexes. Removing overlapping complexes reduces any redundancies in the dataset, enhancing the accuracy and efficiency of the algorithm.

While accuracies are comparable, we note that the RL algorithm achieves faster running time relative to Super.Complex (Table 3). Specifically, the RL algorithm trains for ~ 9 s on one core of a personal computer (M1 chip @ 3.2 GHz), making the training significantly faster than Super. Complex’s training with the AutoML pipeline, which runs for ~ 540 s on 20 cores of a supercomputer (Intel(R) Xeon(R) CPU E5-2699 v3 @ 2.30 GHz). Growing the candidate communities took ~ 300 s when running in parallel across 8 cores (3.2 GHz) for the RL algorithm, compared to ~ 20 s when running in parallel across 72 cores (2.3 GHz) for Super.Complex. This indicates that growing new complexes is also fast in the RL algorithm due to the simple inference using the value function lookup. We employ the four heuristics available in Super.Complex, with default parameters, to find the best one and perform a parameter sweep of 7 thresholds for merging overlaps with each heuristic. In total, we evaluate 28 heuristic-parameter combinations in Super.Complex; the same number of overlap thresholds used in the RL method. Overall, with the best parameters, the RL algorithm took ~ 350 s on the personal computer with 8 cores (note that only the prediction step is parallelized here), compared to Super.Complex which took ~ 650 s on a supercomputer with 72 cores (note that both the learning and the prediction step are parallelized here).

**Table 3:** The RL algorithm achieves a faster running time when compared to Super.Complex

Method	Processor specifications	Training		Prediction		Post-processing		Total time (s)
		No. of cores	Time (s)	No. of cores	Time (s)	No. of cores	Time (s)	
RL Algorithm	M1 chip @ 3.2 GHz	1	9	8	320	1	11	340
Super.Complex	Intel(R) Xeon(R) E5-2699 v3 @ 2.30 GHz	20	540	72	17	1	112	669

The time reported here corresponds to runs using the best overlap threshold found for both methods and using the best heuristic found in the case of Super.Complex

The average time complexity of the prediction phase of the RL algorithm is $O\left( {G^{2} K^{2} S/P} \right)$, where *G* is the average number of nodes in a complex, *K* is the average degree of the network and *S* is the number of seeds chosen (in our experiments, S is the number of nodes in the network). For Super.Complex with all subgraph features, the time complexity of the prediction phase is $O\left( {XG^{4} KS/P} \right)$. We note that the prediction phase of the RL algorithm scales better than that of Super.Complex. This is because the complexity of the subgraph feature extraction step reduces from $O\left( {G^{3} } \right)$ in Super.Complex to $O\left( {GK} \right)$ in the RL algorithm; this reduction happens since the RL algorithm uses only the feature density with a constant model inference time (*X*). The time complexity of the RL training algorithm is $O\left( {G^{3} K^{2} T} \right)$, where *T* is the number of training complexes. In contrast, the training complexity of Super.Complex is $O\left( {G^{3} Tgpm/c} \right)$, where g is the number of generations, p is the population size, m is the number of machine learning models and feature preprocessor types tried, and c is the number of processes on the single compute node running the AutoML step.

We note that the RL algorithm can be especially useful in community detection problems with a small number of known complexes, as demonstrated in our experiments (7 and 132 training complexes in the synthetic and real datasets respectively). Even if the number of known complexes is small, for each complex, the value iteration procedure in the RL algorithm explores several trajectories to learn the complex, incidentally, increasing the size of the training dataset used to learn the complexes. On the other hand, existing supervised community detection methods train on a dataset with a size equal to the number of training complexes. Other benefits of the RL algorithm include the lack of need for extensive hyperparameter tuning and the ability to predict complexes that do not contain smaller complexes. For comparison, in Super.Complex, at each stage of growth in a candidate complex, the pipeline seeks to yield a final protein complex, attempting 4 different heuristics, each with 1–2 hyperparameters. Contrastingly, the RL pipeline learns and traverses the optimal trajectory to find a complex without optimizing for intermediate complexes, and without the need for heuristics, thus saving on searching for parameters in the candidate complex growth step. Thus, the RL algorithm finds the best sequence of steps to grow a complex, while also being efficient.

In summary, relative to more sophisticated supervised ML strategies, the simplicity of the value iteration algorithm and the comparable accuracy along with improved efficiency demonstrates the great potential of the RL algorithm for solving community detection problems.

### The learned clusters suggest functions for uncharacterized proteins

**Fig. 9:**
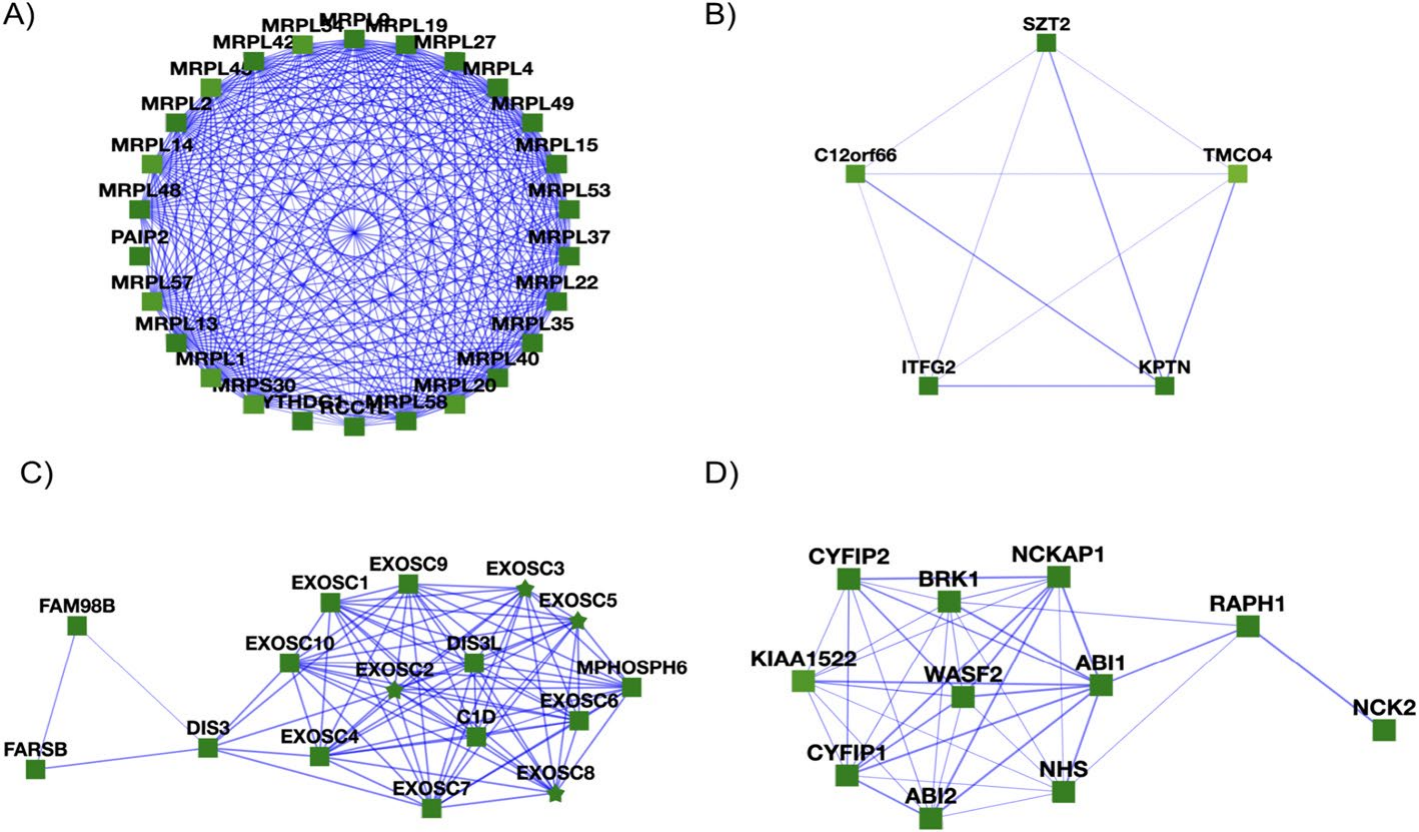
Learned complexes from the RL pipeline. **A** The large (39S) subunit of the mitochondrial ribosome is present in the RL-determined complexes, but broken up into multiple complexes, one of which is shown here. Note the presence of the guanine nucleotide exchange factor RCC1L, which is known to be essential for mitochondrial ribosome biogenesis [30]. **B** RL recapitulates the KICSTOR complex (C12orf66, KPTN, ITFG2, and SZT2), a multiprotein complex known to regulate mTORC1 and cells’ responses to available nutrient levels [31], but finds one additional putative subunit, the uncharacterized transmembrane protein TMCO4. **C** The exosome RNA processing complex is well-reconstructed by RL, with additional interactions observed to the tRNA synthetase FARSB and to FAM98B, a component of tRNA splicing ligase, consistent with possible associations among these systems [35]. **D** The WAVE1/WAVE2 protein complexes, known to regulate actin filament and lamellipodia formation [32, 33], are reconstructed by RL, along with evidence for interaction with the uncharacterized protein KIAA1522. Notably, KIAA1522 was recently suggested by Cho and colleagues to bind WAVE and participate in a community of associated actin-organizing proteins [34]

Importantly, the RL algorithm returns many well-known human protein complexes accurately (as would be expected from the precision measurements on withheld test complexes), several of which are illustrated in Fig. 9.

Moreover, a noteworthy feature of the RL algorithm is that it adds a protein to a complex only if the protein increases or maintains the value function of the complex. For example, this can be seen when building the learned complex in Fig. 9D. When the seed edge is WASF2-ABI1, the algorithm chooses CYFIP1 as its first neighbor to add, resulting in a value function of 0.39. As the algorithm loops through various neighbors, the only neighbors added to the candidate complex are those that maintain the 0.39 value function of the complex. Interestingly, though in most cases the density of the complex is increasing, there are some additions to the complex that decrease the complex density such as when adding NCK2. This is unlike traditional greedy complex detection algorithms that favor higher-density protein complexes. Similarly, this pattern occurs when building the candidate complex with proteins, C15orf41, CDAN1, ASF1A, and HIRA (Fig. 11). Notably, the algorithm also identifies several additional interaction partners and even potential new subunits within these systems, such as, for example, clustering the guanine nucleotide exchange factor RCC1L with proteins of the mitochondrial ribosome large subunit, consistent with a known role for RCC1L in mitochondrial ribosome biogenesis [35]. Similarly, the RL algorithm recapitulates the nutrient-response-related KICSTOR complex (SZT2, KPTN, ITFG2, and C12orf66) [36] on both hu.MAP 1.0 (SZT2, KPTN, ITFG2, TMCO4 and C12orf66) and hu.MAP 2.0 (SZT2, KPTN, ITFG2, TMCO4, BMT2, and KICS2), suggesting the uncharacterized transmembrane protein TMCO4 to be a potential new interaction partner, and it reconstructs the WAVE1/WAVE2 protein complexes, known regulators of actin filament and lamellipodia formation [37, 38] while also suggesting involvement of KIAA1522, consistent with a recent suggestion for its involvement by Cho and colleagues [39]. To investigate the identified complexes interactively, visualizations are available for the 1157 learned complexes on the supporting website (see the Code and data availability section).

**Fig. 10:**
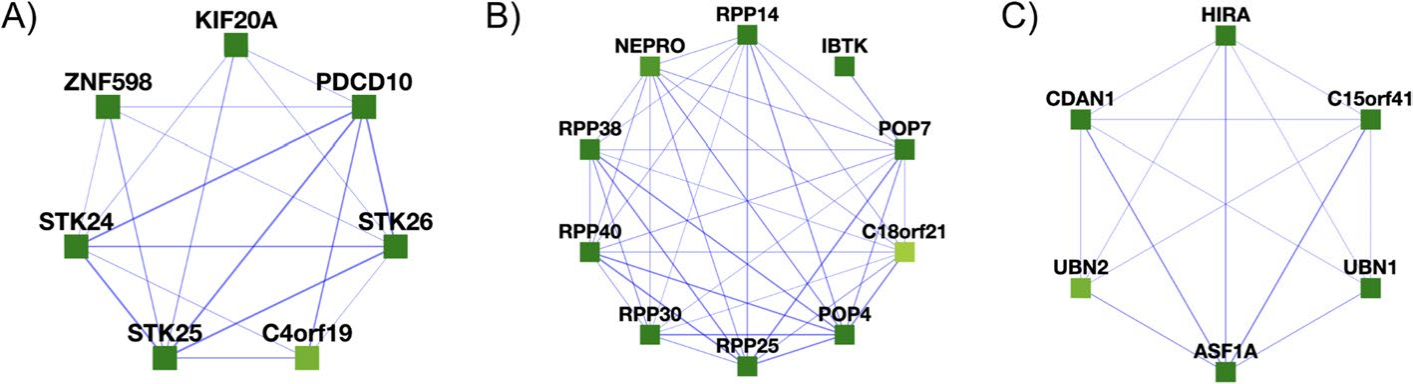
Participation in protein complexes by the uncharacterized proteins C4orf19 and C18orf21 and the minimally characterized protein C15orf41. **A** We find C4orf19 to belong to a larger complex composed of KIF20A, C4orf19, PDCD10, STK25, ZNF598, STK26, and STK24. **B** C18orf21 is found in a complex with 50% similarity to the Rnase/Mrp complex. **C** C15orf41 is found in a complex with 30% similarity to the cytosolic Codanin-1-Asf1-H3.1-histone H4–importin-4 complex

Of particular interest are complexes corresponding to proteins with low annotation scores, as finding the proteins in complexes with better-annotated proteins may help suggest potential functions for these otherwise minimally characterized proteins [40]. We searched specifically for such cases and highlighted complexes with uncharacterized proteins based on available UniProt annotations [41]. Some examples of learned complexes with uncharacterized or minimally characterized proteins are provided in Fig. 10.

For example, in Fig. 10A, C4orf19 (chromosome 4 open reading frame 19) is broadly expressed across human cell types and tissues [42], with high protein levels in the kidney, liver, and GI tract [43, 44] and while little is known about its function, an observed relationship between C4orf19 and colorectal cancer suggests that high expression levels might have some value as a marker for colorectal cancer [45], although elevated C4orf19 expression is also reported to show a favorable association with renal cancer survival [43, 44]. Notably, four of the other proteins in this cluster (PDCD10, STK24, STK25, STK26) are known to associate into a complex with roles in maintaining epithelial integrity [46, 47] and kidney water balance by regulating aquaporin trafficking and abundance in kidney tubule epithelial cells [48], suggesting a potential role for C4orf19 in normal kidney function. As for C4orf19, many of the proteins have been reported as potential biomarkers for bladder, gastric, pancreatic, and colorectal cancers [49–52].

As another example of a minimally characterized protein, C18orf21 (chromosome 18 open reading frame 21) is reported to possibly regulate the Rnase/Mrp complex, a ribonucleoprotein complex involved in RNA processing [53]. Both RL algorithm and Super.Complex concur on a connection for C18orf21 to RNA processing: from the learned complexes of Super.Complex, C18orf21 was found to be a part of a complex with a 50% overlap to the Rnase/Mrp complex, comprising all the proteins found in the RL algorithm’s learned complex (Fig. 10B), adding support for this protein’s possible function in ribonuclease P RNA binding. Further, the RL algorithm learns a similar complex (C18orf21, IBTK, RPP30, POP4, and RPP25L) on hu.MAP 2.0, adding additional support to C18orf21’s function from the learned complexes on hu.MAP 1.0.

Somewhat more information can be gleaned for C15orf41 (chromosome 15 open reading frame 41), which, while minimally characterized, has recently been detected to interact with Codanin-1 (CDAN1) in human cells, and this interaction forms a tight, near stoichiometric complex [54]. Moreover, these studies reveal that mutation of C15orf41 can lead to the development of Congenital Dyserythropoietic type 1 disease (CDA-1) [54]. While its function is unknown, studies have noted a high sequence similarity between C15orf41 and archaeal Holliday junction resolvases, which are DNA repair enzymes that remove Holliday junctions [54], and it has been implicated in erythrocyte differentiation [55]. Its putative interaction partners within the complex (Fig. 10C), HIRA and ASF1A, cooperate to promote chromatin assembly [56], and HIRA, ASF1A, and UBN1/2 form a complex and function in histone deposition of variant H3.3 into chromatin, independent of DNA replication [57]. CDAN1 and C15orf41 mutations lead to similar erythroid phenotypes and they were both eliminated from the same animal taxa, suggesting that these 2 proteins may participate in a shared pathway [58].

**Fig. 11:**
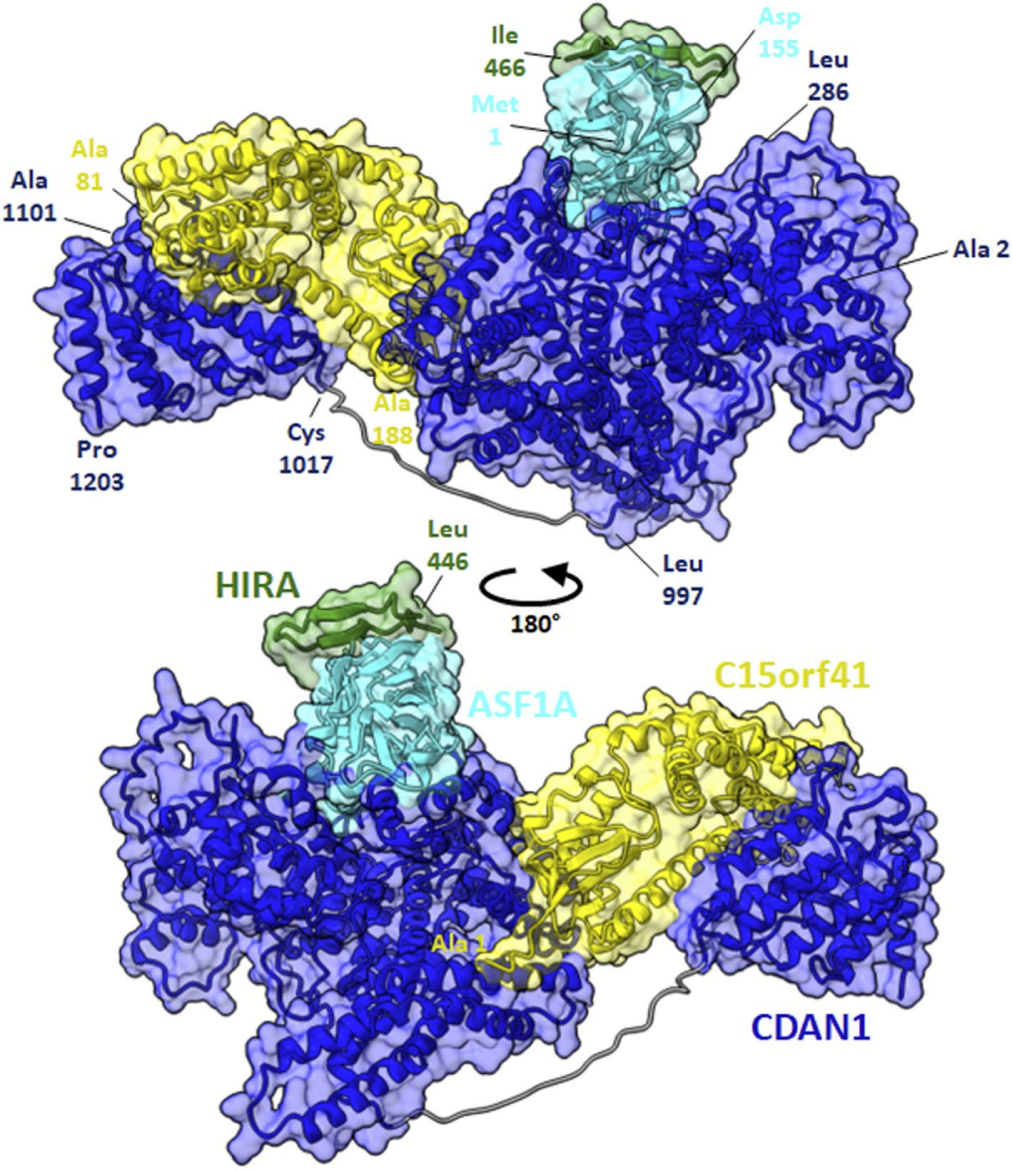
Structural modeling supports C15orf41, CDAN1, ASF1A, and HIRA participating in a large multiprotein complex. Using AlphaFold-multimer, we find that all four proteins can be simultaneously accommodated within a single multiprotein complex, here showing C15orf41’s modeled interaction with CDAN1 residues 2–74 and 286–1203, ASF1A residues 1–155, and HIRA residues 421–479. For illustration purposes, the known crystal structure of HIRA 446–466 [56] has been superimposed onto the AlphaFold model, which is available in full from the supporting GitHub repository with accompanying quality measurements

To obtain more support for the overall physical association of proteins in this cluster, we modeled the 3D structure of the C15orf41-CDAN1 interaction using AlphaFold-Multimer [59], as implemented in Google Colab [60]. The AlphaFold model indicated a high-confidence interface spanning two distinct domains of CDAN1, one contributed from a domain spanning amino acids 1017–1203 and one covering one face of the larger N-terminal domain (2–997) centered on amino acids 427–472 and 843–997; these, in turn, interact with opposing surfaces of C15orf41 (Fig. 11). The predicted structure is consistent with the prior experimental observation that the C-terminal 227 residues of CDAN1 (residues 1000–1227) are critical for the interaction [54]. To investigate the possibility of additional direct interactions between the C15orf41-CDAN1 heterodimer and one or more of the remaining proteins in the cluster, we took advantage of an available X-ray crystal structure that delineated the ASF1A interaction with HIRA residues amino acids 446–466 (PDB entry 2I32) [56] to further evaluate a larger complex. Using AF2-multimer, we modeled C15orf41, CDAN1 residues 2–74 and 286–1203 (omitting the intrinsically disordered segments, as determined by [60]), ASF1A residues 1–155 (omitting the intrinsically disordered tail), and HIRA residues 421–479, a somewhat larger segment known to be critical for the interaction with ASF1A [55]. As illustrated in full in Fig. 11, AlphaFold suggested a binding site for ASF1A distinct from the C15orf41 binding site that, importantly, did not occlude the experimentally determined HIRA binding site, which AlphaFold also recapitulated. Thus, 3D structural modeling confirmed that four of the proteins in this cluster can be accommodated within the same overall multiprotein complex.

Finally, C11orf42 was found as a subunit in a complex (C11orf42, SNX1, SNX5, VPS29, SNX2, COMMD9) that corresponds to a subcomplex of the Retromer or SNX/BAR complex (e.g. as in [61]), with supporting independent evidence from a learned complex from Super.Complex (C11orf42, SNX1, SNX5, and VPS29). This indicates that C11orf42 may be involved in trafficking with Retromer complex proteins, a notion supported by its localization to intracellular vesicles similar in nature to the other proteins in the complex [43, 62–66]. Another example, C16orf91 constituted a complex (C16orf91, UQCC1, COX20, UQCC2) resembling a learned complex from Super.Complex (C16orf91, UQCC1, COX20).

## Conclusions

In conclusion, we asked if reinforcement learning could be applied to learn to walk trajectories on a protein interaction network, and in this way more accurately determine protein complexes. Application of the method to currently available human protein interaction networks performed competitively with other algorithms, with comparable accuracy but notable savings in computational time, and in turn led to clear predictions of protein function and interactions for several uncharacterized human proteins. We could support at least one of these, C15orf41, with independent evidence from 3D structural modeling.

Three main avenues can be explored to improve the RL community detection algorithm: improving the subgraph representations, the RL formulation, and the candidate community search process.

We currently represent a subgraph by a single feature, its density, which gives a problem formulation with a small state space. While performance may be negatively impacted, to improve accuracy, more subgraph features could be included in addition to density. Examples of other subgraph features that could be added include edge weight statistics, node clustering coefficient statistics, and degree correlation statistics. However, as the number of features increases, the state space increases exponentially. For instance, if we incorporate 18 topological features with a discretization of each feature into 10 bins, we increase the number of states to $1{0}^{18}$. Different sample-based reinforcement learning methods could be applied to address this challenge and potentially give more accurate results.

The RL formulation could be modified to better accommodate overlapping community detection, by giving a positive reward if, while growing a seed node, a neighbor is present in any of the training complexes that can be built, rather than considering only one training community at a time. Note that in this scenario, the reward for a neighbor can change dynamically based on the possible communities that can be built from that step. Due to the dynamic rewards that need to be computed for each neighbor at each iteration, by checking whether the new subgraph is a part of any of the training communities, computational time would increase significantly compared to the current static reward system, however, it may improve accuracy. Also, different rewards and discount factors can be experimented with in the training phase of the algorithm.

Finally, in the candidate community search process, there may be scenarios where there are multiple highest-scoring neighbors to add at an iteration in the growth process of a subgraph. Currently, we have only added one of the highest-scoring neighbors to grow the complex. Each of the other highest-scoring neighbors can be added as well to grow complexes we may have missed in the current method.

## Supplementary Information

**Additional file 1:** This file contains the following supplementary figures and tables. **Figure S1.** Convergence of other scores from the training RL algorithm. **Table S1.** RL algorithm performance on training and testing toy complexes. **Table S2.** RL algorithm performance on training and testing hu.MAP 1.0 complexes. **Table S3.** RL algorithm performance on hu.MAP 2.0 complexes.

## Data Availability

Code, available on GitHub repository: https://github.com/marcottelab/RL_complex_detection. Data: All learned complexes from hu.MAP 1.0 with their corresponding scores: https://marcottelab.github.io/RL_humap_prediction/humap/res_pred_names.txt. All learned complexes from hu.MAP 2.0 with their corresponding scores: https://marcottelab.github.io/RL_humap_prediction/humap2/res_pred_names_humap2.txt. Interactive visualizations of results on hu.MAP 1.0 and hu.MAP 2.0: To investigate the complexes identified by the RL algorithm interactively, visualizations are available for the learned complexes on hu.MAP 1.0 and hu.MAP 2.0 on this website: https://marcottelab.github.io/RL_humap_prediction/. The website also provides the functionality of sorting complexes and proteins by their annotation score and the number of interactions with SARS-CoV-2 proteins [67]. Structural model for the C15orf41, CDAN1, ASF1A, and HIRA heterotetramer, with sequences and assessments of model quality, can be downloaded from: https://github.com/marcottelab/RL_humap_prediction/ (Folder—CDIN1_CDAN1_2-74_286-1203_ASF1A_1-155_HIRA_421-479_1ModelRelaxed.zip).
